# In Vivo Evaluation of the Skin Tensile Strength by the Suction Method: Pilot Study Coping with Hysteresis and Creep Extension

**DOI:** 10.1155/2013/841217

**Published:** 2013-08-05

**Authors:** Gérald E. Piérard, Sébastien Piérard, Philippe Delvenne, Claudine Piérard-Franchimont

**Affiliations:** ^1^Laboratory of Skin Bioingineering and Imaging (LABIC), Department of Clinical Sciences, Liège University, 4000 Liège, Belgium; ^2^Telecommunications and Imaging Laboratory INTELSIG, Montefiore Institute, Liège University, 4000 Liège, Belgium; ^3^Department of Dermatopathology, Unilab Lg, Liège University Hospital, 4000 Liège, Belgium

## Abstract

From an engineering standpoint, both the skin and subcutaneous tissue act as interconnected load-transmitting structures. They are subject to a variety of intrinsic and environmental influences. Changes in the cutaneous viscoelasticity represent an important aspect in a series of skin conditions. The aim of this work was to explore the methodology of biomechanical measurements in order to better appreciate the evolution and severity of some connective tissue diseases. The Cutometer MPA 580 (C+K electronic) was used in the steep and progressive suction procedures. Adapting measurement modalities was explored in order to mitigate any variability in data collection. The repeat steep suction procedure conveniently reveals the creep phenomenon. By contrast, the progressive suction procedure highlights the hysteresis phenomenon. These viscoelastic characteristics are presently described using the 2 and 4 mm probes on normal skin and in scleroderma, acromegaly, corticosteroid-induced dermatoporosis, and Ehlers-Danlos syndrome. The apposition of an additional outer contention on the skin altered differently the manifestations of the creep extension and hysteresis among the tested skin conditions. Any change in the mechanical test procedure affects the data. In clinical and experimental settings, it is mandatory to adhere to a strict and controlled protocol.

## 1. Introduction

Measurements of a number of physical parameters characterizing human skin have been attempted over the recent decades. A diversity of devices assessing skin viscoelasticity were used both in vitro and in vivo [1, 2]. They proved to be useful tools for scientists and medical practitioners [3, 4]. Over a large part of the body, the overall viscoelastic behaviour of the skin primarily depends on the skin connective tissue (SCT) structures present in both the dermis and the subcutis, with minimal contribution from the epidermis [5–7]. 

The suction method is one of the most widely used approach for determining some of the biomechanical characteristics of human skin in health and disease [8–17]. The progressive suction mode with a stress-versus-strain graphic recording is a convenient way in this endeavour [9–11]. In this procedure, a progressive increase in stress suction for a defined period of time is followed by a symmetrical rate of suction release. During the whole process, skin deformation defined as the strain is recorded. Typically, viscoelastic materials exhibit nonlinear stress-versus-strain properties [1, 2, 9, 17]. The hysteresis loop represents the area delimited by the two curves representing the loading and relaxation phases, respectively.

Another measurement modality corresponds to the steep suction mode with a stress-versus-time graphic representation [7, 10, 11]. A single or a series of steep changes in suction and relaxation are applied to the skin. The progressive rise in maximum skin deformation reached in the successive cycles defines the creep extension.

The purpose of this pilot study was to revisit the hysteresis loop and the creep extension as observed using a specific time-honored noninvasive suction method routinely applicable in clinical settings. Assessments were performed on normal skin as well as in specific conditions associated with viscoelastic changes in the skin. These disorders included acroscleroderma, Ehlers-Danlos syndrome (EDS), corticosteroid-induced dermatoporosis [18], and untreated acromegaly. We show that both steep and progressive procedures are convenient complementary modalities for assessing skin viscoelasticity. Two types of analytical data including creep extension and hysteresis loop generated by the dual different procedures should be taken in consideration for rating skin viscoelasticity changes in connective tissue disorders.

## 2. Patients and Methods

### 2.1. Design

The study was approved by the Ethic Committee of the University Hospital, and it was performed in accordance with the Declaration of Helsinki. A total of 120 Caucasian subjects of both genders, aged 24–48 years, were enrolled. The volunteers signed an informed consent after the entire procedure of the study had been fully explained. The study was performed between Fall 2007 and Spring 2012.

 A total of 60 healthy subjects (32.1 ± 4.9 years, M/F: 27/33) formed the normal reference group. Four other groups of 15 subjects each had been diagnosed with systemic scleroderma (29.8 ± 6.4 years, M/F: 6/9), hypermobile EDS (35.2 ± 3.8 years, M/F: 10/5), corticosteroid-induced dermatoporosis (37.4 ± 4.6 years, M/F: 9/6), and untreated low-grade acromegaly (28.6 ± 7.0 years, M/F: 8/7).

### 2.2. Procedure

**Figure 1:**
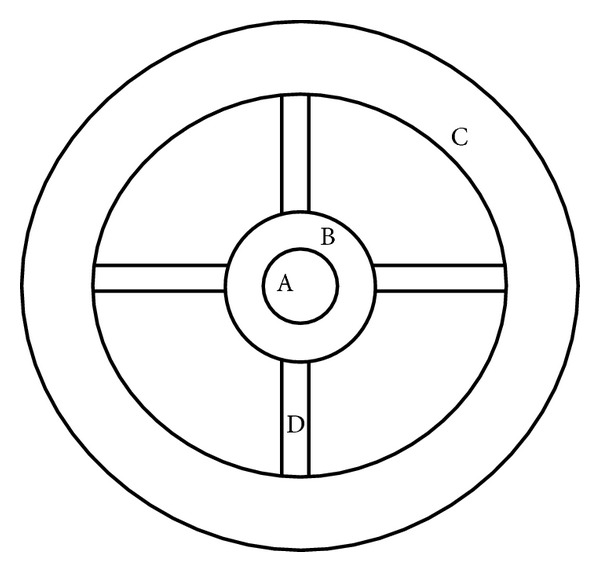
Schematic drawing of the probe positioning onto the skin: (A) probe aperture; (B) probe guard wall; (C) outer guard ring; (D) adhesive tape.

Both the Cutometer SM 474 and MPA 580 versions (C+K electronic, Cologne, Germany) are computer-assisted suction devices. Each of them was equipped with two hollow probes centered by a 2 or 4 mm diameter aperture, respectively. Each handheld probe was maintained on the skin surface under constant pressure guaranteed by a built-in spring. Upon suction, the skin surface was pulled upwards inside the probe opening by the applied negative pressure. The vertical skin deformation was measured optically with a 0.01 mm accuracy. The assessments were performed on the midvolar aspect of both forearms. On the left forearm, the skin adjacent to each probe was grossly maintained in place by the guard wall of the probe. On the right forearm, an additional concentric 55 mm diameter steel guard ring was affixed to the skin by a double-side adhesive film. In addition, adhesive tapes (acrylate paper type or silicone tape) were placed in a crosswise pattern between the outer guard ring and the probe (Figure 1). The two probes were successively applied 3.5 cm apart from each other. The device was used under two distinct modalities, namely, the steep and progressive modes as previously described [5, 19, 20].

**Figure 2:**
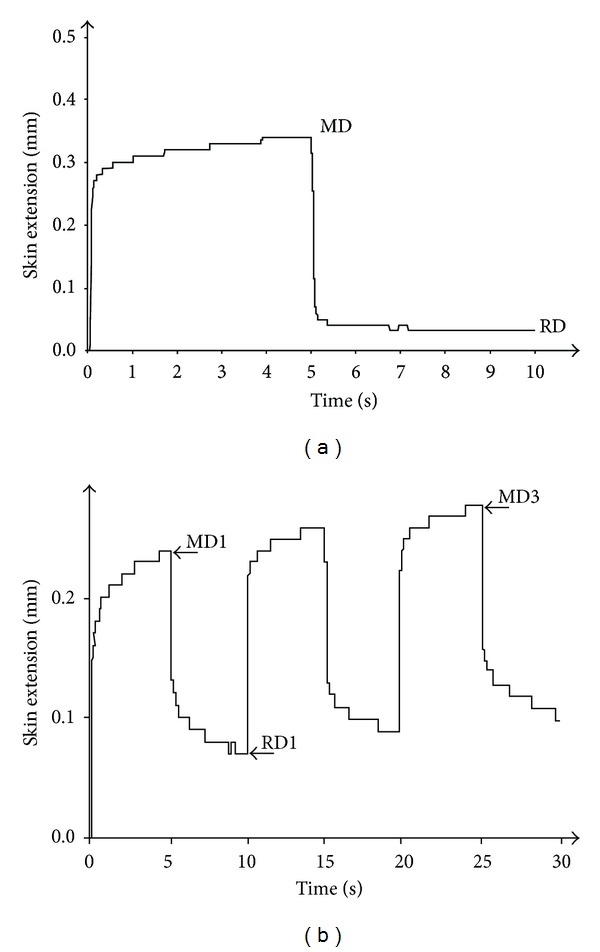
Strain-versus-time curve obtained under the steep mode procedure. A 500 mbar suction applied for 5 s followed by a relaxation time of 5 s. The skin extensibility characterizes the maximum deformation (MD) and the residual deformation (RD): (a) single cycle on normal skin, (b) triple cycle on normal skin.

In the steep suction mode, the vertical skin deformation was recorded as a function of time. For a given probe aperture, the level of steep negative pressure (500 mbar), the duration (5 s) of both the suction time (stress on) and relaxation time (stress off), and the number of measurement cycles (1, 3, and 5) were selected (Figures 2(a) and 2(b)). The chosen parameters under the steep mode of measurement were the maximum deformation (MD), the residual deformation (RD), and the viscoelastic creep (ΔMD) between the first and either the third MD (ΔMD3) or the fifth (ΔMD5) deformation cycle (Figure 2(b)). The corresponding ΔRD3 and ΔRD5 were similarly calculated. 

**Figure 3:**
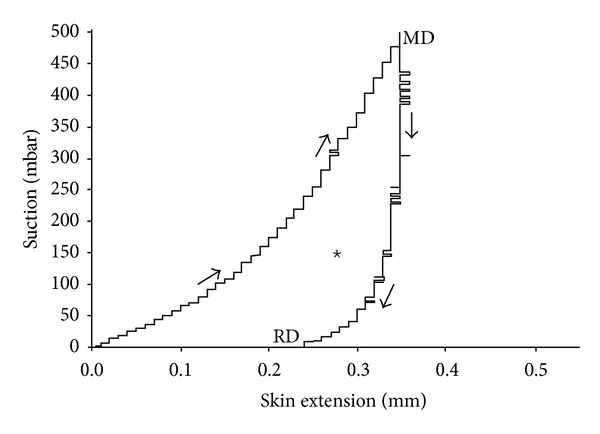
Stress-versus-strain curve obtained under the progressive suction procedure. A progressive linear increase in suction of 25 mbar/s for 20 s followed by a relaxation recovery at the same rate. The maximum deformation (MD) and the residual deformation (RD) are recorded. Hysteresis (★) is the area delimited by the suction-relaxation curves.

In the progressive suction mode, the vertical skin deformation was measured as a function of the progressive negative pressure applied for a 20 s-linear increase in suction (25 mbar/s) followed by a similar rate of linear decrease in suction force for a 20 s-relaxation period (Figure 3). The nonlinear stress-strain curves on suction and relaxation were not superposed. During the 20 s-relaxation period, the values of strain did not return to zero, and the intercept of the curve on the strain axis defined the residual deformation (RD). The area delimited between the two curves corresponded to the hysteresis loop. It was measured in arbitrary units using computerized image analysis of the graphs (MOP Videoplan Kontron, Eching, Germany).

Sets of single steep and progressive suction procedures were performed at a given day. One week later, series of repetitive measurements (3 or 5) were performed under the steep suction modality.

### 2.3. Statistical Analysis

Magnitude, spread, and symmetry of the data were assessed using the Shapiro-Wilks test. Data were expressed as means and standard deviations or as medians and range according to the data distribution. Statistical comparisons were performed using variance analysis. A *P* value < 0.05 was considered significant.

## 3. Results

Data about MD and RD are presented in Table 1. The steep and progressive suction modalities globally showed congruent information, and some data were significantly different between selected skin conditions.

In the steep suction mode using the 4 mm aperture probe, the comparison with normal skin showed that MD was significantly increased (*P* < 0.05) in hypermobile EDS and decreased (*P* < 0.01) in acroscleroderma. In the same procedure, RD was significantly increased (*P* < 0.05) in dermatoporosis and decreased (*P* < 0.05) in hypermobile EDS. By contrast, no significant changes were yielded between the disorders when using the 2 mm aperture probe.

In the progressive suction mode using the 4 mm aperture probe, the comparison with normal skin revealed a significant (*P* < 0.01) MD decrease in both acroscleroderma and acromegaly. In the same conditions, RD was significantly decreased in hypermobile EDS (*P* < 0.01). Conversely, RD was significantly increased (*P* < 0.05) in acroscleroderma, dermatoporosis, and acromegaly. The same procedure using the 2 mm probe revealed significant (*P* < 0.05) RD increases in dermatoporosis and acromegaly.

Both the creep extension and hysteresis loop were observed approximately at the same magnitude on normal skin as well as in the four pathologic conditions considered in this study (Table 2).

### 3.1. Creep Extension

**Figure 4:**
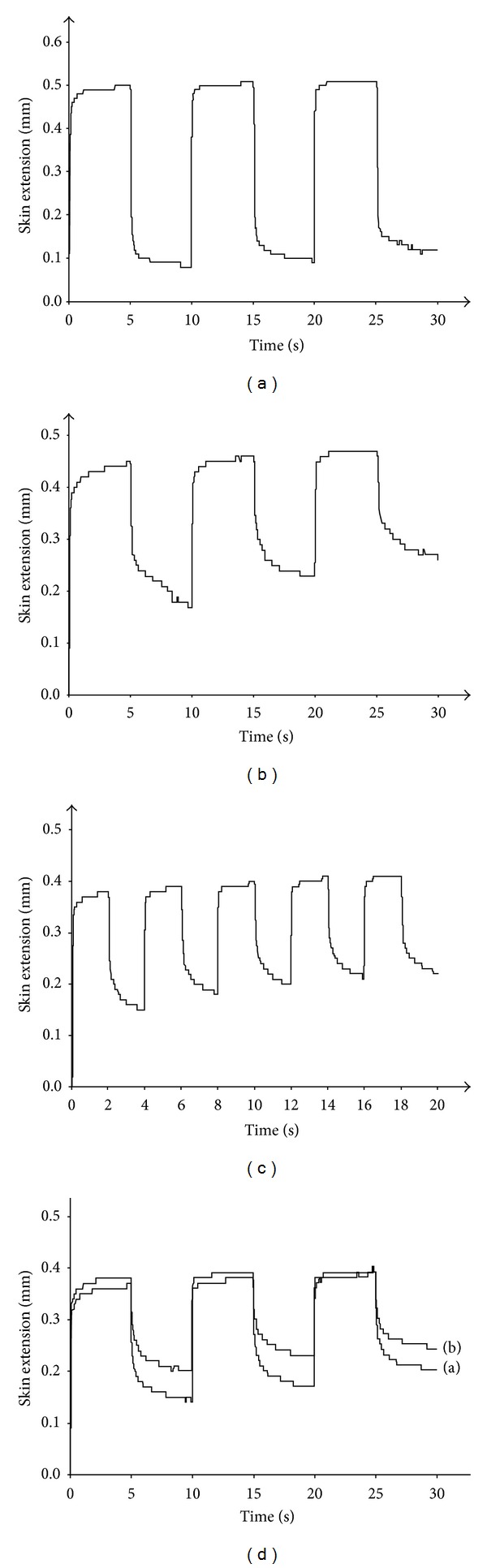
Repetitive strain-versus-time curve obtained under the steep mode procedure. (a) Hyperextensible and hyperelastic skin. (b) Skin with increased resistance to recovery after skin deformation. (c) Linear increase in MD and RD with successive cycles. (d) Aspects of 2 series of 3 cycles of suction. MD values are similar, while the RD are higher in the second series (b) than in the initial one (a).

The repeat steep test modality revealed the creep extension (ΔMD3 and (ΔMD5) presenting as a progressive but moderate MD increase during successive suction cycles (Table 2). Of note, the successive RD values increased more largely than the corresponding MD. Hence, ΔRD3 and ΔRD5 were repeatedly superior to the corresponding ΔMD. The ΔMD was more prominent with the larger probe aperture size (Table 2). The various skin conditions did not influence the magnitude of the creep extension (*P* > 0.05). The combination of prominent MD and low RD values (Figure 4(a)) was commonly associated with both minimal ΔMD and ΔRD. By contrast, when MD was less intense and RD was raised (Figure 4(b)), both ΔMD and ΔRD were increased. In any circumstance, the raises in both MD and RD were linear during the successive suction cycles (Figure 4(c)). Typically, the repetition of successive triple suction cycles was associated with minimal ΔMD, although ΔRD was going up (Figure 4(d)). 

**Figure 5:**
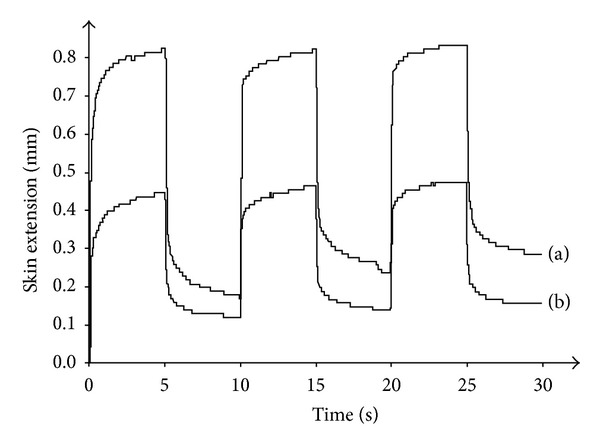
Repetitive stress-versus-time curves without (a) and with (b) outer contention showing a prominent effect on MD, a moderate effect on RD, and a minimal effect on ΔMD and ΔRD.

The outer contention ring contributed to reduce the creep extension particularly in loose skin (*P* < 0.05). In case of large MD at the regular procedure, the application of the outer contention resulted in a reduction (*P* < 0.05) of this parameters (Figure 5). RD was reduced at a lower extent (*P* > 0.05).

### 3.2. Hysteresis

The hysteresis loop was disclosed under the progressive suction modality. For any given suction stress, strain was always superior during the relaxation phase than during the increasing suction phase. At the selected rate of stress application (25 mbar/s for 20 s) on normal skin, the progressive skin deformation under suction was discretely curved or nearly linear, irrespective of the probe size and the presence or absence of the outer contention ring. By contrast in similar test conditions, the relaxation curve showed larger bulging. Typically, the initial portion of the relaxation curve was characterized by plasticity corresponding to a near absence or discrete reduction in the strain deformation. By contrast, the rate of strain reduction down to RD was maximized during the late portion of the relaxation phase. 

In different skin conditions, some differences were yielded in the hysteresis loop according to the probe size (Table 3). The 2 mm aperture probe without any outer contention yielded a significant (*P* < 0.05) hysteresis decrease in hypermobile EDS and increase in dermatoporosis. The 4 mm aperture probe yielded a significant (*P* < 0.01) hysteresis decrease in hypermobile EDS. In each condition, the interindividual range of data was quite large with much overlap between the groups of subjects.

The combination of the 2 mm aperture probe with the outer guard ring yielded significant (*P* < 0.05) hysteresis decrease in hypermobile EDS and increase in dermatoporosis compared to normal skin. The combination of the 4 mm aperture probe with the outer guard ring yielded a significant increase in hysteresis in acroscleroderma (*P* < 0.01) and acromegaly (*P* < 0.05), whereas it was significantly (*P* < 0.01) decreased in hypermobile EDS.

## 4. Discussion

The preponderant viscoelastic properties of skin are governed by SCT components [6, 17]. Both the dermis and hypodermis are characterized by their own intimate structures whose tensile functions are balanced to adequately respond to the casual mechanical demands [21]. It is acknowledged that a series of physiopathological variables alter the viscoelasticity of the whole skin [10, 17, 20, 22, 23]. Accordingly, the assessment of skin viscoelasticity provides incentives for progress in skin care management.

The Cutometer is a time-honored and widely spread device. The suction force, its rate of application, and the duration of suction are controlled [3, 12, 13]. Clearly, the repeatability and reproducibility of measurements are optimal on inert material (rubber, silicone sheet, …). However, in vivo repetitive measurements on human skin show some variations in data collection according to age, body location, and SCT disorders [17, 24, 25]. The Cutometer generates two types of analytical data according to the steep and progressive suction applications [9, 17]. This report describes the effects of controlled measuring procedures in health and SCT diseases.

 In most biomechanical study designs, the crude information received from an experiment is the relationship linking any applied force to the relative deformation over time. Basically, in controlled in vitro studies, the term stress corresponds to the ratio between the suction and the test area of skin in a plane at right angles to the direction of the force [17]. The term strain represents the ratio between tissue elongation and its original length. Therefore, it is dimensionless, since measured as millimetres per millimetre. These definitions are altered in the in vivo Cutometer application as the negative pressure applied to the skin corresponds to the notion of stress, irrespective of the size of the probe aperture, and strain is simply the vertical elevation of skin. 

During the suction procedure, some increased elongation takes place under stable or repeat tractions and is not completely reversed within a short time in the absence of compressive force [10, 17]. This means that RD is typically present and possibly interferes with subsequent testing at the same site during the next few minutes. These changes in mechanical characteristics are referred to as the creep, viscous extension, or viscous slip. Accordingly, the procedure of skin preconditioning is achieved by applying a series of stresses to the tissue before measuring its subsequent viscoelasticity. During the creep phenomenon, any positive ΔMD probably reflects a progressive sliding motion of collagen bundles inside the SCT. Although the creep extension (ΔMD) remains limited with regard to the MD magnitude, the ΔRD increases at a larger extent probably due to a progressive limitation in the elastic recovery following a change in the collagen bundle arrangement. Any ΔRD results from a mitigated function of the network of elastic fibres pulling back the fibrous collagen bundles to the rest position with maximum entropy.

Clearly, the Cutometer in its clinical applications is not a diagnostic tool but rather a functional assessor for SCT disorders. For a given pathological condition, the interindividual variations expressed by each parameter are quite large. However, the patterns of associated viscoelastic changes are consistent in each of the considered disorders [5, 9, 19, 26–29]. It is noteworthy that data yielded by any given probe aperture do not predict data gained by other probes. In our experience, the 4 mm probe is more informative than the 2 mm probe in SCT disorders. Data collected by the steep suction procedure do not predict the information gained by the progressive suction procedure. Both procedures are complementary.

Unsurprisingly, the outer contention exerts its maximum effect on loose skin. This procedure is responsible for a plasticity phenomenon. It limits the phase of skin elastic extension but exerts little effect on the viscous extension. Hence, in our experience an additional outer contention is only useful in case of SCT looseness.

In summary, both the steep and progressive suction procedures are convenient complementary modalities for assessing skin viscoelasticity. The creep extension and the hysteresis loop should be taken in consideration for rating skin viscoelasticity changes in connective tissue disorders.

## Figures and Tables

**Table 1:** Steep and progressive suction procedures without outer contention. Median and range values of viscoelastic parameters related to one single suction cycle according to the two recording modalities for the two probe aperture diameters (AD 2 mm and AD 4 mm) on normal skin and connective tissue disorders.

Skin condition	MD (mm)		RD (mm)	
	AD 2 mm	AD 4 mm	AD 2 mm	AD 4 mm
Steep modality				
Normal	0.28	0.42	0.08	0.14
	(0.12–0.33)	(0.21–0.59)	(0.05–0.16)	(0.08–0.22)
Scleroderma	0.19	0.25**	0.10	0.13
	(0.09–0.24)	(0.10–0.37)	(0.04–0.18)	(0.08–0.18)
Ehlers-Danlos	0.30	0.61*	0.03	0.09*
	(0.15–0.50)	(0.27–0.89)	(0.02–0.12)	(0.04–0.13)
Dermatoporosis	0.24	0.45	0.13	0.31*
	(0.14–0.35)	(0.18–0.63)	(0.09–0.23)	(0.16–0.35)
Acromegaly	0.18	0.30	0.10	0.20
	(0.09–0.30)	(0.23–0.39)	(0.08–0.20)	(0.13–0.17)

Progressive modality				
Normal	0.21	0.38	0.07	0.09
	(0.11–0.26)	(0.28–0.51)	(0.05–0.12)	(0.07–0.13)
Scleroderma	0.17	0.23**	0.13	0.17*
	(0.05–0.21)	(0.10–0.31)	(0.07–0.18)	(0.06–0.20)
Ehlers-Danlos	0.26	0.47	0.06	0.05**
	(0.08–0.43)	(0.39–0.87)	(0.04–0.15)	(0.03–0.08)
Dermatoporosis	0.24	0.42	0.21*	0.34*
	(0.16–0.43)	(0.32–0.65)	(0.15–0.33)	(0.24–0.36)
Acromegaly	0.19	0.27**	0.16*	0.21*
	(0.13–0.31)	(0.17–0.40)	(0.14–0.22)	(0.12–0.24)

Compared to normal; *P < 0.05; **P < 0.01.

**Table 2:** Steep suction procedure. Median values of parameters defining the creep extension in the repeat stress-versus-time recording modality (3 and 5 cycles) for different probe aperture diameters (AD 2 mm and AD 4 mm) using a 500 mbar depression for 5 s performed with (w) and without (w/o) outer contention on normal skin and connective tissue disorders. The differences in maximum deformation after 3 and 5 cycles (ΔMD3, and ΔMD5, and the corresponding residual deformations (ΔRD3 and ΔRD5) were recorded.

Skin condition	ΔMD3 (%)				ΔMD5 (%)				ΔRD3 (%)				ΔRD5 (%)			
	AD 2 mm		AD 4 mm		AD 2 mm		AD 4 mm		AD 2 mm		AD 4 mm		AD 2 mm		AD 4 mm	
	w	w/o	w	w/o	w	w/o	w	w/o	w	w/o	w	w/o	w	w/o	w	w/o
Normal	3	3	5	5	4	5	7	5	5	6	6	6	6	6	7	7
Scleroderma	1	1	2	2	1	1	2	2	2	2	3	4	3	4	3	4
Ehlers-Danlos	1	1	1	2	3	4	5	5	3	4	5	5	4	5	5	5
Dermatoporosis	1	1	4	5	2	2	4	5	2	2	4	5	5	5	9	5
Acromegaly	2	2	5	3	2	2	2	2	2	2	5	4	2	3	2	3

**Table 3:** Progressive suction procedure performed with (w) and without (w/o) outer contention. Median and range value of hysteresis (arbitrary units) obtained with the two probe aperture diameters (AD 2 mm and AD 4 mm) on normal skin and connective tissue disorders.

Skin condition	AD 2 mm		AD 4 mm	
	w	w/o	w	w/o
Normal	99 (76–118)	97 (72–136)	114 (86–127)	128 (89–154)
Scleroderma	111 (95–114)	115 (93–120)	127** (110–139)	143 (123–158)
Ehlers-Danlos	68* (56–95)	66* (62–99)	70** (66–104)	75** (68–111)
Dermatoporosis	122* (101–134)	133* (95–148)	138 (109–143)	156 (104–169)
Acromegaly	111 (94–126)	106 (82–133)	132* (101–158)	130 (115–179)

Compared to normal; *P < 0.05; **P < 0.01.
